# Scaling the EVERREST of severe, early-onset fetal growth restriction

**DOI:** 10.1172/JCI173563

**Published:** 2023-09-15

**Authors:** Emily J. Su

**Affiliations:** Division of Reproductive Sciences/Division of Maternal-Fetal Medicine, Department of Obstetrics and Gynecology, University of Colorado School of Medicine, Aurora, Colorado, USA.

## Abstract

Severe, early-onset fetal growth restriction is a leading cause of medically indicated preterm birth and substantially increases the risk for perinatal death or disability. No treatments exist to improve fetal growth or safely prolong pregnancy. Furthermore, wide-ranging phenotypes limit the accurate prediction of pregnancy outcome. In this issue of the *JCI*, Spencer and colleagues combine a discovery-science approach with ultrasound parameters to identify the most discriminative models for predicting either the primary outcome of fetal or neonatal death, or a secondary outcome of death or delivery at 28 weeks of gestation or earlier. Their findings can better individualize patient counseling but, just as compellingly, provide the capacity to identify those pregnancies that are at such considerable risk as to justify enrollment in paradigm-shifting interventional trials that are in the pipeline.

## Consequences of severe, early-onset fetal growth restriction

The phenotype of fetal growth restriction (FGR), defined as the inability of a fetus to achieve its growth potential, is wide ranging. In its mildest form, delivery occurs at term gestation and carries few risks. In contrast, severe, early-onset FGR is a leading cause of medically indicated preterm birth, with substantial risk for adverse perinatal outcomes as a result of prematurity that are further worsened by exposure to an abnormal in utero environment (1, 2). These outcomes include stillbirth, neonatal death, and among survivors, high risk for chronic lung disease, necrotizing enterocolitis, and neurodevelopmental deficits.

**Figure 1:**
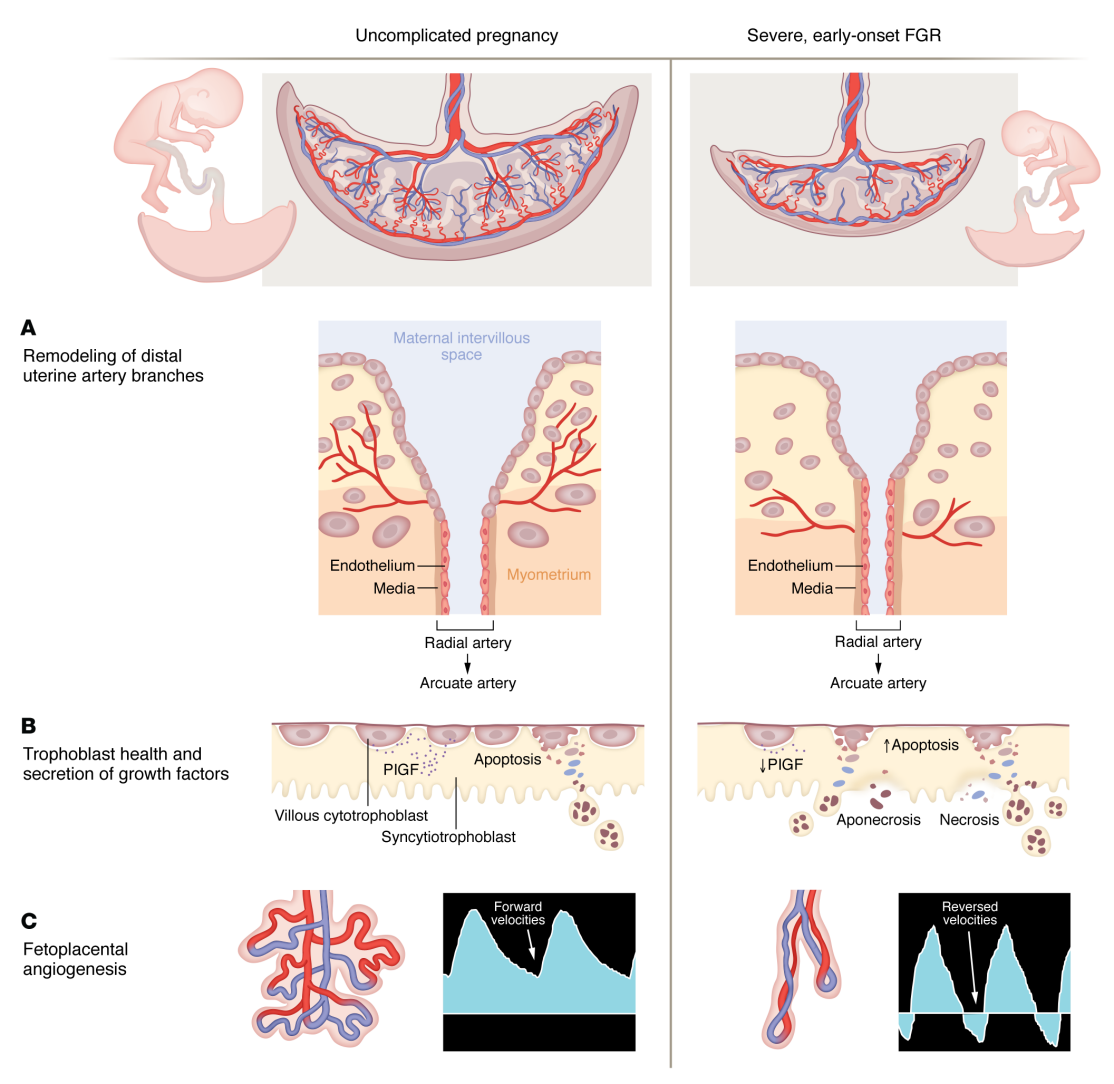
Pathologic mechanisms of severe, early-onset FGR influence key prognostic indicators. (**A**) Inadequate remodeling of the uterine spiral artery and its distal branches early in pregnancy results in malperfusion of the maternal intervillous space. (**B**) Maternal vascular malperfusion contributes to reduced cytotrophoblast proliferation, diminished syncytial fusion, and increased release of necrotic and apoptotic trophoblastic material. The unhealthy trophoblast layer secretes insufficient levels of growth factors such as PlGF into the maternal vascular compartment and provides inadequate surface area for maternal-fetal exchange. (**C**) As gestation progresses, concomitant impairments in fetoplacental angiogenesis result in villous vessels that are abnormally elongated and poorly branched. This vascular deficiency not only contributes to improper maternal-fetal exchange but also leads to abnormally elevated fetoplacental vascular resistance. Clinically, umbilical artery Doppler waveforms show deterioration, where forward velocities that normally occur during fetal cardiac diastole progressively diminish, becoming absent or even reversed.

Accounting for up to 30% of all FGR cases, early-onset FGR most commonly arises secondarily to placenta-mediated causes. Deficient transformation of the distal branches of the uterine spiral arteries in early pregnancy leads to maternal vascular malperfusion (3). As gestation progresses, inadequate expansion of the villi and fetoplacental vessels results in decreased surface area for maternal-fetal exchange and elevated fetoplacental vascular resistance, respectively, the latter of which is clinically ascertained by umbilical artery Doppler interrogation (Figure 1) (4). The resultant hypoxemia and hypoxia and increased cardiac afterload lead to fetal cardiac deterioration, and in the absence of delivery, result in stillbirth (5).

Beyond immediate consequences, exposure to an insufficient in utero environment begets developmental programming that increases the risk for long-term diseases. In the case of FGR, these conditions include coronary heart disease, hypertension, stroke, and metabolic syndrome (6). For instance, it has been shown that reduced intrauterine oxygen and nutrient supply disrupt cardiomyocyte growth and fiber architecture (7). Paired with increased placental resistance and fetal cardiac afterload, the developing myocardium undergoes cardiac remodeling that persists through life. Furthermore, these individuals are more likely to experience microvascular endothelial dysfunction and increased aortic intima-media thickness (7), precursors to hypertension and atherosclerosis. In turn, these cardiometabolic disorders predispose those who were growth restricted themselves to developing complications such as FGR in their own future pregnancies, resulting in multigenerational effects on long-term health arising from a single dysfunctional placenta.

## Diagnostic and management dilemmas

For technical and practical reasons, fetuses are diagnosed as growth restricted when their estimated fetal weight (EFW) is below the tenth percentile for gestational age (GA). This criterion does not account for fetuses that are achieving their intrinsic growth prospects and are just constitutionally small, nor does it identify fetuses with EFWs at or above the tenth percentile but that are not reaching their inherent growth potential. While misidentification becomes less of an issue when using an EFW cutoff of less than the third percentile, it remains possible that fetuses in this category can still be misdiagnosed as abnormally small when they are, in fact, meeting their innate growth trajectories. Thus, the ability to accurately discriminate between normal and pathologic growth is needed in order to appropriately identify pregnancies that require heightened surveillance and to decrease any possibility of unindicated, iatrogenic preterm delivery as a result of false-positive antenatal testing.

In fetuses at high risk for growth restriction, Doppler interrogation of the umbilical artery is customarily performed as a way to help identify pathologic growth restriction while also assessing fetal well-being. However, the validity of measurements depends on the portion of the cord that is sampled and fetal physiologic parameters such as heart rate and movement. There is also evidence to support some prognostic utility of Doppler assessment of the middle cerebral artery, ductus venosus, umbilical vein, and maternal uterine arteries, but these have not been universally adopted as standard of care for various reasons outside the scope of this Commentary. Furthermore, maternal serum biomarkers that are typically obtained for purposes of aneuploidy screening may suggest increased risks for pathologic fetal growth but are certainly not diagnostic or prognostic. Hence, identification of highly sensitive and specific biomarkers of growth restriction that wield additional capacity to predict outcomes is sorely needed.

The tenets of managing pregnancies complicated by severe, early-onset FGR have also been frustratingly static. Timing delivery to balance the risks of intrauterine fetal demise or irreversible injury with the risks of prematurity remains the cornerstone of care in these pregnancies. However, clinical trials investigating the timing of delivery for participants who predominantly or uniformly exhibited severe FGR found no differences in overall survival (8–10). Measures aimed at FGR prevention such as aspirin or heparin (including low-molecular-weight heparin) have shown inconsistent and modest reductions in prevalence, at best (11). Moreover, interventional trials of phosphodiesterase type 5 inhibitors (e.g., sildenafil) also have not demonstrated efficacy in overall outcome, improvement of fetal growth, or safe prolongation of pregnancy in severe FGR (12–14). Thus, more sophisticated and effective modalities to manage and treat these pregnancies are critically needed.

In this issue of the *JCI*, Spencer and colleagues leveraged a discovery-science approach combined with sonographic parameters to identify and validate prognostic markers at the time of severe FGR diagnosis (15). This study incorporated participants from the EVERREST Prospective Study, which was a multicenter prospective cohort study taking place in four European tertiary referral centers (16). Individuals with singleton pregnancies with an EFW of less than 600 g and below the third percentile for GA between 20 0/7 and 26 6/7 weeks’ gestation were eligible. Maternal serum at the time of enrollment was subjected to immunoassays, Olink proximity extension assays, and liquid chromatography with tandem mass spectrometry. Sonographic measurements were obtained, including biometry and Doppler velocimetry of the umbilical artery, uterine artery, middle cerebral artery, ductus venosus, and umbilical vein. In total, 63 participants were included in the discovery set, with another 60 individuals comprising the validation set, and final validated models incorporated estimates from both groups.

As determined by the discovery set, the best sonographic predictor of the primary outcome of fetal or neonatal death was the EFW *z* score, a measure of standard deviation from the EFW mean. In contrast, the umbilical artery Doppler category yielded the highest prediction for the secondary outcome of death or delivery at 28 0/7 weeks of gestation or less. After combining maternal serum biomarker data from the discovery and validation sets, placental growth factor (PlGF) was identified as being the most strongly associated with the primary outcome of fetal or neonatal death and the secondary outcome of death or delivery at or before 28 0/7 weeks of gestation. Receiver operating characteristic (ROC) curves of combined data sets showed that, while the EFW *z* score, umbilical artery category, and GA at enrollment were most predictive of fetal or neonatal death (AUC, 0.91), the most discriminative model for death or delivery at or prior to 28 0/7 weeks was PlGF in combination with the umbilical artery category (AUC, 0.89).

## Umbilical artery Doppler and PlGF in severe FGR

Umbilical artery Doppler velocimetry reflects fetoplacental vascular resistance. In uncomplicated pregnancies with appropriate fetal growth, vascular resistance gradually decreases with advancing GA. This progression allows for forward flow through the umbilical arteries throughout the fetal cardiac cycle. When placental vascular development is substantially disrupted, forward flow in the umbilical arteries during fetal diastole progressively decreases, potentially further deteriorating into absent or even reversed end-diastolic velocities and putting substantial strain on the fetal heart.

PlGF, a member of the VEGF family, is a proangiogenic protein that is highly expressed in trophoblasts and secreted into the maternal blood as early as 11 to 13 weeks of gestation. Normally, concentrations progressively increase, peaking at about 28 weeks and remaining stable until 36 weeks, when concentrations then slowly decline (3). In placenta-mediated FGR, PlGF secretion is impaired (Figure 1), which contributes to the shift in balance of factors toward an antiangiogenic state. While low levels of PlGF in the maternal circulation carry some predictive value in the development of FGR (17–19), its utility as a prognostic indicator of pregnancy outcome in severe FGR, especially one identified through a discovery-based approach, had not been investigated until Spencer et al. (15).

## Clinical and research implications

This compelling study by Spencer and colleagues highlights several important considerations with respect to severe, early-onset FGR (15). On a cellular and molecular level, the identification of PlGF as the maternal serum protein most highly associated with the primary (and one secondary) outcome suggests that mechanisms regulating uterine and placental angiogenesis are potentially viable targets for the treatment of severe FGR. From a more immediate clinical standpoint, these findings may be used to further bolster individualized patient counseling. However, the most exciting impact of the study by Spencer et al. (15) is the potential ability to identify which severe, early-onset FGR pregnancies are at high enough risk to warrant enrollment into innovative and paradigm-shifting interventional trials, such as the EVERREST trial (20). This phase I/IIa trial aims to deliver maternal uterine artery VEGF gene therapy to individuals with an extreme phenotype of severe, early-onset FGR who are most likely to benefit from this intervention and for whom the standard treatment of preterm delivery is either too risky or futile. Preclinical studies suggest that local enhancement of angiogenesis and vascular function improve fetal growth, which would also allow for safe prolongation of pregnancy (20). The potential impact of this unprecedented trial that is in the pipeline cannot be overemphasized, and the findings of Spencer et al. (15) lay additional groundwork to continue scaling the EVERREST of severe, early-onset FGR.

## Version 1 09/15/2023

Electronic publication

## References

[1] Lingam I Neonatal outcomes following early fetal growth restriction: a subgroup analysis of the EVERREST study.. Arch Dis Child Fetal Neonatal Ed.

[2] Malhotra A (2019). Neonatal morbidities of fetal growth restriction: pathophysiology and impact. Front Endocrinol (Lausanne).

[3] Zur RL (2020). The placental basis of fetal growth restriction. Obstet Gynecol Clin North Am.

[4] Sun C (2020). The placenta in fetal growth restriction: What is going wrong?. Placenta.

[5] Nawathe A, Lees C (2017). Early onset fetal growth restriction. Best Pract Res Clin Obstet Gynaecol.

[6] Barker DJ (2006). Adult consequences of fetal growth restriction. Clin Obstet Gynecol.

[7] Crispi F (2018). Long-term cardiovascular consequences of fetal growth restriction: biology, clinical implications, and opportunities for prevention of adult disease. Am J Obstet Gynecol.

[8] GRIT Study Group (2003). A randomised trial of timed delivery for the compromised preterm fetus: short term outcomes and Bayesian interpretation. BJOG.

[9] Lees CC (2015). 2 year neurodevelopmental and intermediate perinatal outcomes in infants with very preterm fetal growth restriction (TRUFFLE): a randomised trial. Lancet.

[10] Thornton JG (2004). Infant wellbeing at 2 years of age in the Growth Restriction Intervention Trial (GRIT): multicentred randomised controlled trial. Lancet.

[11] Nawathe A, David AL (2018). Prophylaxis and treatment of foetal growth restriction. Best Pract Res Clin Obstet Gynaecol.

[12] Groom KM (2019). STRIDER NZAus: a multicentre randomised controlled trial of sildenafil therapy in early-onset fetal growth restriction. BJOG.

[13] Pels A (2020). Maternal sildenafil vs placebo in pregnant women with severe early-onset fetal growth restriction: a randomized clinical trial. JAMA Netw Open.

[14] Sharp A (2018). Maternal sildenafil for severe fetal growth restriction (STRIDER): a multicentre, randomised, placebo-controlled, double-blind trial. Lancet Child Adolesc Health.

[15] Spencer R (2023). Maternal PlGF and umbilical Dopplers predict pregnancy outcomes at diagnosis of early-onset fetal growth restriction. J Clin Invest.

[16] Spencer R (2017). EVERREST prospective study: a 6-year prospective study to define the clinical and biological characteristics of pregnancies affected by severe early onset fetal growth restriction. BMC Pregnancy Childbirth.

[17] Benton SJ (2016). Placental growth factor as a marker of fetal growth restriction caused by placental dysfunction. Placenta.

[18] Shinar S (2021). Placental growth factor as a diagnostic tool for placental mediated fetal growth restriction. Pregnancy Hypertens.

[19] Tong S (2019). Blood-based biomarkers in the maternal circulation associated with fetal growth restriction. Prenat Diagn.

[20] David AL (2017). Maternal uterine artery VEGF gene therapy for treatment of intrauterine growth restriction. Placenta.

